# The human gut microbiota is associated with host lifestyle: a comprehensive narrative review

**DOI:** 10.3389/fmicb.2025.1549160

**Published:** 2025-06-23

**Authors:** Qin Zeng, Xianqiong Feng, Yanling Hu, Shaoyu Su

**Affiliations:** ^1^Department of Pediatrics Nursing, West China Second University Hospital, Sichuan University, Chengdu, China; ^2^Key Laboratory of Birth Defects and Related Diseases of Women and Children (Sichuan University), Ministry of Education, Chengdu, China; ^3^Department of Nursing, West China Hospital, Sichuan University, Chengdu, China; ^4^Nursing Key Laboratory of Sichuan Province, West China School of Nursing, Sichuan University, Chengdu, China

**Keywords:** gut microbiome, precision nutrition, circadian biology, exercise immunology, metabolic health

## Abstract

**Background:**

The gut microbiota serves as a critical interface between lifestyle factors and host physiology. Despite extensive research on individual domains including diet, sleep, and exercise, an integrated understanding of their synergistic effects on microbial communities remains incomplete. This knowledge gap limits our ability to develop targeted microbiome-based interventions for metabolic and immune-related disorders.

**Methods:**

To address this gap, we conducted a comprehensive evaluation of peer-reviewed literature from 2000 to present, identified through systematic searches of PubMed, Web of Science, and Scopus using key terms related to gut microbiota and lifestyle interventions. Our analysis focused on studies incorporating microbiome profiling techniques, controlled lifestyle interventions, and multi-omics data integration. The review prioritized mechanistic insights from both clinical and preclinical investigations while critically assessing methodological approaches across the field.

**Results:**

High-fiber dietary patterns consistently promoted the abundance of beneficial, short-chain fatty acid-producing bacteria, though with notable inter-individual variation. Circadian rhythm disruption was associated with reduced microbial diversity and expansion of pro-inflammatory bacterial taxa, paralleling increases in systemic inflammation markers. Athletic populations demonstrated unique microbial signatures characterized by enhanced metabolic potential, with distinct taxonomic profiles emerging across different sport disciplines.

**Conclusion:**

This work synthesizes current evidence into a novel framework for understanding lifestyle-microbiota interactions, while identifying key challenges in study design and data interpretation. We propose standardized methodological approaches for future investigations and outline translational strategies for personalized microbiota modulation. These insights advance the potential for targeted microbial interventions to optimize metabolic and immune health outcomes.

## 1 Introduction

The human gut microbiota constitutes a complex, dynamic ecosystem comprising bacteria, archaea, fungi, viruses, and eukaryotes that collectively encode >3 million genes—150-fold more than the human genome (Qin et al., 2010). This “second genome” plays pivotal roles in nutrient metabolism (Sonnenburg and Bäckhed, 2016), immune system maturation (Zheng et al., 2020), and neuroendocrine signaling through the gut-brain axis (Cryan et al., 2019). Mounting evidence from large-scale initiatives like the Human Microbiome Project (Qin et al., 2010) and MetaHIT (Arumugam et al., 2011) highlights the role of gut microbial dysbiosis in human health. This imbalance has been linked to the pathogenesis of various conditions, from metabolic disorders such as obesity and T2DM (Wu et al., 2021) to neurological diseases like Parkinson’s and autism spectrum disorders (Lynch and Pedersen, 2016). Particularly compelling are recent findings showing that gut microbiota composition can predict individualized glycemic responses to foods (Ben-Yacov et al., 2023), suggesting its potential as a therapeutic target. Furthermore, recent studies have highlighted the significant impact of diet, exercise, and other lifestyle factors (e.g., sleep) on gut bacterial composition. According to a 2023 review by Pedroza Matute and Iyavoo (2023), adjustments to these lifestyle elements hold potential as effective avenues for personalized interventions aimed at enhancing gut health and overall well-being. However, the precise mechanisms by which modifiable lifestyle factors influence microbial community structure and function remain incompletely characterized, creating an urgent need for systematic synthesis of current evidence. While psychological stress, alcohol consumption, and medication use also modulate gut microbiota, this review focuses on diet, sleep, and exercise due to their direct modifiability and robust evidence base.

To address this gap, we systematically evaluated peer-reviewed English-language literature (2000–present). Our searches in PubMed, Web of Science, and Scopus employed Boolean logic targeting: (“gut microbiota” OR “gut microbiome”) AND (“diet” OR “nutrition”), (“circadian rhythm” AND “microbiota”), and (“exercise” AND “microbial diversity”). Excluded studies primarily focused on non-modifiable factors (e.g., genetics) or lacked microbial profiling data. Our analysis prioritized: (i) Randomized controlled trials (RCTs) with microbial profiling (16S rRNA sequencing, metagenomics); (ii) Longitudinal cohort studies incorporating multi-omics data; (iii) Mechanistic animal studies employing germ-free or gnotobiotic models; (iv) Seminal reviews and meta-analyses to synthesize evolving theoretical frameworks. Key foundational works (David et al., 2014; Turnbaugh et al., 2009) were considered alongside cutting-edge research [e.g., fecal microbiota transplantation studies (Ianiro et al., 2022)] to provide both historical context and contemporary perspectives.

This review makes three novel contributions to the field. First, we present a critical appraisal of how distinct dietary patterns (Mediterranean, plant-based, Western) differentially modulate microbial diversity and functional capacity, with particular attention to the role of microbiota-accessible carbohydrates (Deehan et al., 2020). Second, we synthesize emerging evidence for circadian misalignment-induced dysbiosis and its metabolic consequences (Cheng et al., 2021), proposing testable hypotheses about the gut microbiota’s role in sleep disorder pathophysiology. Finally, we evaluate dose-dependent effects of exercise on microbial short-chain fatty acids (SCFAs) production (Mailing et al., 2019) and identify promising avenues for athlete microbiota optimization. By integrating findings across these domains, we highlight understudied interactions between lifestyle factors and propose a framework for personalized microbiota modulation strategies.

## 2 Gut microbiota in systemic diseases

In the cardiovascular system, a multitude of diseases are associated with gut microbiota. Recent studies have established a strong link between gut microbiota dysbiosis and hypertension pathogenesis. Clinical observations reveal that hypertensive patients consistently show reduced gut microbial diversity, characterized by significant depletion of beneficial bacteria such as *Akkermansia muciniphila* and *Faecalibacterium prausnitzii* (Li et al., 2017). This causal relationship is further supported by experimental evidence demonstrating that fecal microbiota transplantation from hypertensive donors to germ-free mice can directly elevate blood pressure (Yan et al., 2020). Mechanistic investigations indicate that gut dysbiosis promotes increased intestinal permeability and subsequent lipopolysaccharide (LPS) translocation, which triggers systemic inflammation and contributes to vascular stiffness (Luo et al., 2023). Deficiency of short-chain fatty acids, particularly butyrate, has been shown to impair baroreceptor sensitivity and disrupt blood pressure regulation (Muralitharan et al., 2025). Additionally, emerging evidence highlights the critical role of gut microbiota-derived trimethylamine-N-oxide (TMAO) in atherosclerosis pathogenesis (Ma et al., 2022).

Furthermore, within the digestive system, the gut microbiota plays a pivotal role in maintaining gastrointestinal homeostasis, with dysbiosis implicated in various digestive disorders. In inflammatory bowel disease (IBD), patients exhibit reduced microbial diversity, with depletion of anti-inflammatory bacteria like *Faecalibacterium prausnitzii* and overgrowth of pro-inflammatory *Escherichia coli* strains (Sharma et al., 2025). Fecal microbiota transplantation (FMT) has shown remarkable efficacy in *Clostridioides difficile* infection by restoring microbial balance (Baunwall et al., 2020). Mechanistically, gut dysbiosis disrupts mucosal barrier function through altered tight junction proteins (occludin, ZO-1), while microbial metabolites like butyrate regulate intestinal immunity via HDAC inhibition and Treg cell induction (Di Vincenzo et al., 2024). Emerging evidence also links specific microbial signatures (e.g., *Fusobacterium nucleatum* enrichment) to colorectal carcinogenesis through Wnt/β-catenin pathway activation, highlighting the microbiota’s dual role in digestive health and disease pathogenesis (Mondal et al., 2025).

**Figure 1:**
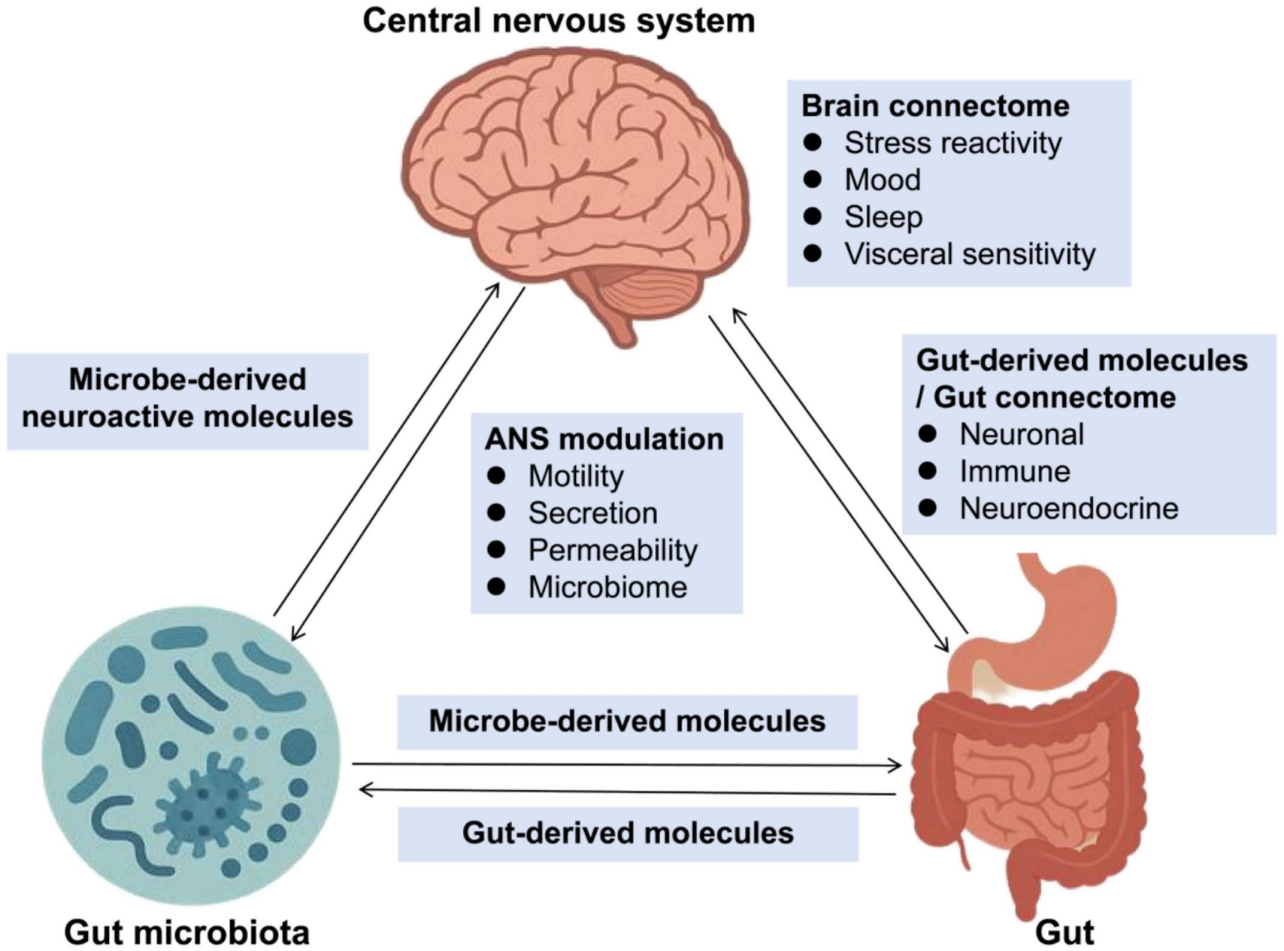
Gut-brain-microbiota axis. ANS, Autonomic Nervous System. This diagram illustrates the complex interactions between the gut microbiota, gut-derived molecules, and the central nervous system (CNS), highlighting the role of the brain connectome and ANS modulation. The gut-brain-microbiota axis plays a crucial role in maintaining homeostasis and influencing various physiological and psychological functions.

Additionally, in the Nervous system, emerging evidence demonstrates a bidirectional communication between the gut microbiota and the central nervous system, termed the gut-brain axis, which plays a pivotal role in neurological health (Figure 1). In Parkinson’s disease (PD), patients exhibit decreased *Prevotella* and increased Enterobacteriaceae abundance, correlating with motor symptom severity (Bi et al., 2022; Blommer et al., 2023). Notably, fecal microbiota transplantation from PD patients to mice induces α-synuclein aggregation and motor deficits (Munoz-Pinto et al., 2024; Sampson et al., 2016). Mechanistically, gut dysbiosis promotes neuroinflammation via microglial activation through LPS-TLR4 signaling (Niño et al., 2018), while microbial metabolites like SCFAs modulate blood–brain barrier integrity (Parker et al., 2020). Similarly, in Alzheimer’s disease (AD), reduced microbial diversity and elevated *Escherichia/Shigella* are associated with amyloid-β deposition (Li et al., 2019).

**Figure 2:**
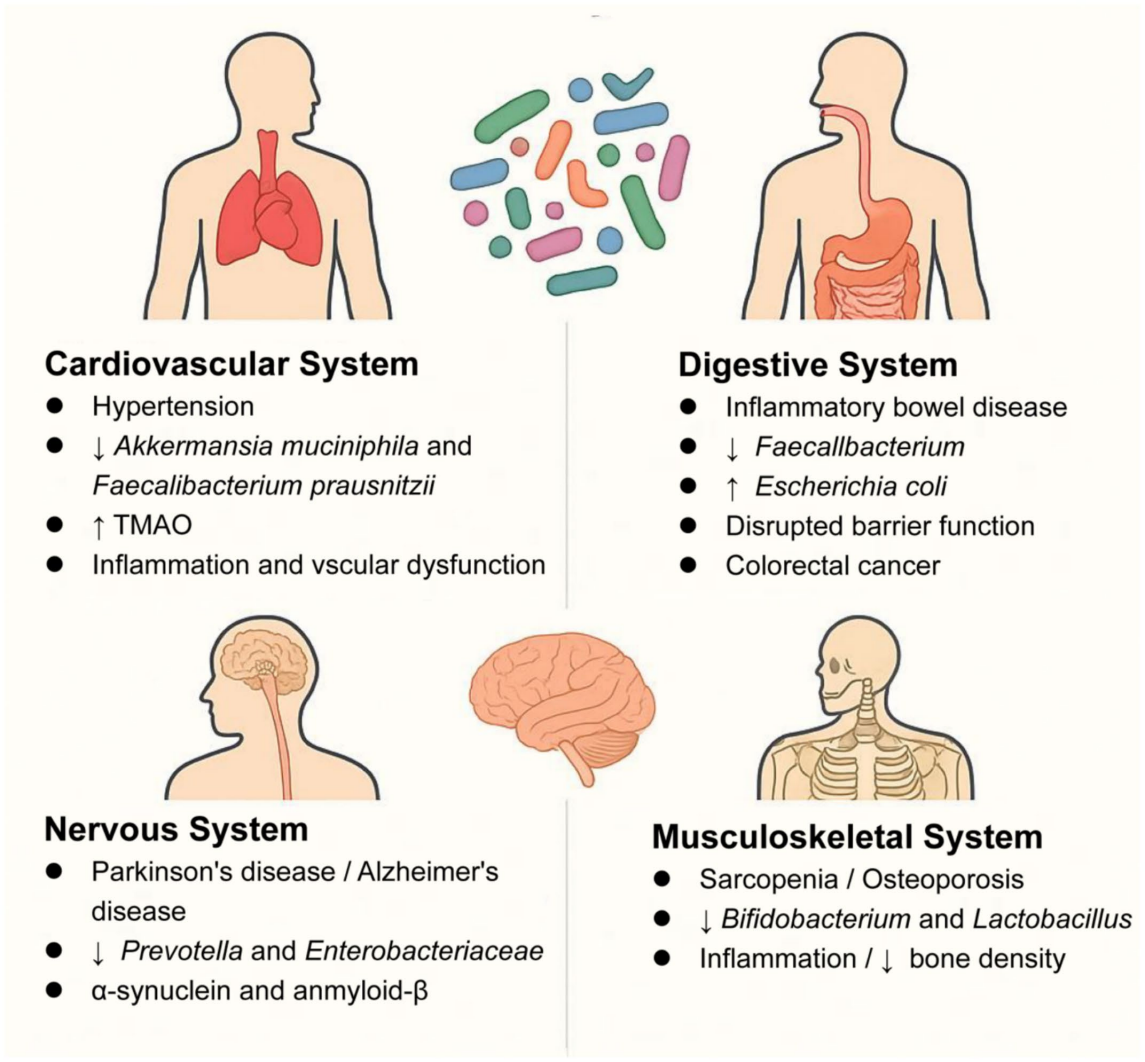
Key microbiota alterations in systemic diseases: ↓ beneficial taxa (*Akkermansia*), ↑ pathogens (*Escherichia coli*). ↑ indicates increase, ↓ indicates decrease; TMAO, trimethylamine N-oxide.

Moreover, in the musculoskeletal system, emerging evidence highlights the critical role of gut microbiota in musculoskeletal health. In sarcopenia, elderly patients exhibit reduced gut microbial diversity, particularly with depletion of *Bifidobacterium* and *Faecalibacterium prausnitzii* (Rashidah et al., 2022). Notably, fecal microbiota transplantation from young donors to aged mice restores muscle mass and strength (Kim et al., 2022), demonstrating a causal link between gut microbiota and sarcopenia. Mechanistically, gut dysbiosis drives systemic inflammation (elevated IL-6/TNF-α) and accelerates muscle protein degradation (Mancin et al., 2023). Conversely, butyrate-producing taxa (e.g., *Roseburia*, *Eubacterium*) enhance mitochondrial function through AMPK activation (Kundu et al., 2019). Similarly, in osteoporosis, postmenopausal women show decreased *Lactobacillus* abundance correlated with lower bone mineral density (Jansson et al., 2019). The gut microbiota-disease relationships across major physiological systems are summarized in Table 1 and Figure 2.

**Table 1:** Gut microbiota-disease associations across physiological systems.

System/disease	Key microbial alterations	Mechanistic pathways	Clinical/experimental evidence
Cardiovascular	↓ *Akkermansia muciniphila*	LPS-induced inflammation	FMT transfers hypertension phenotype
Hypertension	↓ *Faecalibacterium prausnitzii*	Butyrate deficiency	
		TMAO production	
Digestive	↓ *Faecalibacterium prausnitzii*	Tight junction disruption	FMT efficacy in *C. difficile* infection
IBD	↑ *Escherichia coli*	Wnt/β-catenin activation	
Colorectal cancer	↑ *Fusobacterium nucleatum*		
Nervous	↓ *Prevotella*	LPS-TLR4 microglial activation	FMT induces α-synuclein pathology
Parkinson’s disease	↑ Enterobacteriaceae	SCFA-mediated BBB regulation	
Alzheimer’s disease	↑ *Escherichia/Shigella*		
Musculoskeletal	↓ *Bifidobacterium*	IL-6/TNF-α elevation	FMT restores muscle mass in aged mice
Sarcopenia	↓ *Faecalibacterium prausnitzii*	AMPK pathway modulation	
Osteoporosis	↓ *Lactobacillus*		

↑ indicates increase, ↓ indicates decrease; TMAO, trimethylamine N-oxide; SCFA, short-chain fatty acids; FMT, fecal microbiota transplantation.

In addition to being associated with various system diseases in the human body, the gut microbiota is also linked to the host’s lifestyle factors, such as diet, sleep, and exercise.

## 3 The correlation between gut microbiota and host lifestyle

### 3.1 Diet and gut microbiota interactions

#### 3.1.1 Dietary patterns

Dietary habits profoundly shape gut microbiota composition, with distinct microbial signatures emerging across major dietary regimes. Contemporary research highlights three predominant patterns—the Western diet, plant-based diets, and the Mediterranean diet—each exhibiting unique impacts on microbial ecology and host health. The Western diet, characterized by excessive intake of processed foods, saturated animal fats, and refined sugars coupled with low fiber consumption, drives gut dysbiosis through multifactorial mechanisms. Clinical evidence demonstrates significant reductions in microbial diversity and depletion of beneficial SCFA-producing taxa such as *Faecalibacterium prausnitzii*, alongside expansion of pro-inflammatory Enterobacteriaceae and pathobionts like *Clostridium difficile* (Du et al., 2025; Zeng et al., 2022). This dietary pattern elevates circulating TMAO levels by 2.5-fold through microbial choline metabolism, correlating with atherosclerotic plaque formation (Ma et al., 2022), while concurrently decreasing colonic butyrate production by 40–60% compared to fiber-rich diets, thereby impairing epithelial barrier integrity (Sánchez-Tapia et al., 2020). Recent metabolomics analyses further reveal that diet-induced depletion of *Faecalibacterium* decreases colonic butyrate synthesis by 58%, directly impairing mitochondrial β-oxidation in enterocytes (Benjamin et al., 2022). However, the metabolomics analysis in 2025 further revealed that the decrease in *Faecalibacterium prausnitzii* caused by diet would lead to a 58% reduction in colonic butyrate synthesis, directly impairing the mitochondrial beta-oxidation function of intestinal epithelial cells (Münte and Hartmann, 2025). Animal models further reveal that high-fat components selectively enrich bile acid-transforming *Bilophila wadsworthia*, exacerbating colitis via TH17-mediated inflammation (Reynolds et al., 2017), and downregulate tight junction proteins facilitating lipopolysaccharide (LPS) translocation and systemic endotoxemia (Thaiss et al., 2016).

In contrast, plant-based and Mediterranean diets enhance microbial diversity and metabolic homeostasis. High-fiber plant-based regimens enrich *Prevotella-dominant* enterotypes and fiber-degrading specialists such as *Xylanibacter*, driving SCFA production through cross-feeding networks (De Filippo et al., 2010; Portincasa et al., 2022). Within high-fiber plant-based regimens, resistant starch further induces strain-level specialization in *Bifidobacterium adolescentis*, enhancing amylolytic activity while competitively excluding *Clostridium perfringens* (Wang et al., 2025; Zhao et al., 2024; Zhao et al., 2025). The Mediterranean diet synergizes olive oil polyphenols (e.g., hydroxytyrosol) with complex carbohydrates, elevating *Bifidobacterium* abundance and reducing inflammatory markers such as C-reactive protein and interleukin-6 (Haskey et al., 2023; Perrone and D’Angelo, 2025). Long-term adherence to this diet increases fecal butyrate concentrations by 25–30% through *Roseburia-mediated* fermentation, correlating with improved insulin sensitivity (D’Archivio et al., 2022). These findings collectively underscore the critical role of dietary patterns in modulating gut microbial ecosystems, with profound implications for metabolic and inflammatory health outcomes.

#### 3.1.2 Specific dietary components

Key dietary constituents differentially modulate microbial communities through targeted mechanisms. Non-digestible carbohydrates, particularly soluble fiber, serve as keystone substrates for saccharolytic taxa, with soluble fiber-derived butyrate upregulating claudin-1 expression and suppressing NF-κB activation via histone deacetylase (HDAC) inhibition (Cuevas-Sierra et al., 2024). In contrast, insoluble fiber accelerates intestinal transit, reducing pathogenic overgrowth through mechanical clearance (De Filippo et al., 2010; Portincasa et al., 2022). Saturated fats induce *Bilophila*-dominated dysbiosis, activating NLRP3 inflammasomes and increasing inflammatory bowel disease risk, while concurrently reducing *Lactobacillus* abundance and impairing secondary bile acid metabolism (Cani et al., 2007; Henao-Mejia et al., 2012; Sánchez-Tapia et al., 2020). Among micronutrients, olive oil phenolics such as oleuropein inhibit *Fusobacterium nucleatum* biofilm formation and downregulate Wnt/β-catenin signaling in colorectal carcinogenesis (D’Archivio et al., 2022). Similarly, vitamin D insufficiency correlates with *Lactobacillus* depletion and compromised IgA-mediated mucosal immunity (Zeevi et al., 2015). These findings collectively illustrate how specific dietary components orchestrate microbial dynamics, with profound implications for gut homeostasis and disease susceptibility. The impacts of various dietary patterns on gut microbial composition and functional outcomes are summarized in Table 2.

**Table 2:** Effects of dietary patterns on gut microbiota composition and functional outcomes.

Diet type	Key microbial changes	Mechanisms/health implications
High-animal-protein	↑Firmicutes, Enterobacteriaceae, Proteus ↓*Bacteroides*, *Lactobacillus*, *Rosebacillus*	↑Inflammation, metabolic dysfunction, LPS, Chronic low-grade inflammation, Metabolic disorders ↓SCFAs levels
Plant-based	↑*Prevotella*, *Xylanibacter*, *Prevotella*	↑Anti-inflammatory, Insulin sensitivity, SCFAs levels
Mediterranean	↑Firmicutes, *Prevotella*, *Bifidobacterium*	↑Anti-inflammatory, SCFAs levels

↑ indicates increase, ↓ indicates decrease; SCFA, short-chain fatty acids.

#### 3.1.3 Controversies and emerging frontiers

Despite robust evidence linking dietary patterns to microbial alterations, significant controversies persist. While plant-based diets are consistently associated with *Prevotella* enrichment, methodological limitations challenge interpretability, including small sample sizes [e.g., Garcia-Mantrana et al., *n* = 27 (Garcia-Mantrana et al., 2018)] that limit generalizability, cross-sectional designs unable to establish causality, and conflicting outcomes across studies. For instance, high-animal-protein diets variably correlate with Firmicutes abundance (de Wit et al., 2012; Du et al., 2025), and Mediterranean diets show inconsistent effects on α-diversity despite *Bifidobacterium* enrichment (De Filippis et al., 2016; Nagpal et al., 2018). These discrepancies likely stem from methodological heterogeneity, such as divergent dietary assessment tools (e.g., food frequency questionnaires vs. controlled feeding studies) and sequencing platforms (e.g., 16S rRNA vs. shotgun metagenomics) (Armet et al., 2022; Ross et al., 2024; Wilson et al., 2020). Additionally, host-specific confounders, including baseline microbiota composition, genetic polymorphisms, and unmeasured lifestyle factors, contribute to these inconsistencies (Baldi et al., 2024; Diacova et al., 2025). To address these gaps, future studies should prioritize longitudinal designs, standardized protocols, and multi-omics integration (metagenomics, metabolomics, proteomics).

Emerging research extends diet-microbiota interactions to circadian regulation, with SCFAs such as butyrate modulating core clock genes (e.g., Bmal1, Per2) in intestinal epithelial cells, thereby synchronizing host metabolic rhythms and glucose homeostasis (Cheng et al., 2021; Choi et al., 2021). This intersection of dietary habits, microbial ecology, and chronobiology illuminates novel pathways for metabolic disease pathogenesis. Additionally, a bidirectional relationship exists between sleep architecture and gut microbiota: chronic sleep disruption reduces *Faecalibacterium* abundance while elevating pro-inflammatory taxa like Enterobacteriaceae, whereas microbial metabolites (e.g., serotonin precursors) reciprocally regulate sleep quality—a dynamic interplay explored in subsequent sections.

### 3.2 Sleep and gut microbiota interactions

Growing evidence demonstrates a bidirectional relationship between gut microbiota imbalance and sleep disturbances, though current findings indicate correlation rather than causation (Nagpal et al., 2018). The host’s circadian rhythm and gut microbiota exhibit reciprocal regulation, where chronic sleep disruption alters microbial composition and function (Choi et al., 2021; Segers and Depoortere, 2021; Triplett et al., 2020).

Clinical studies consistently report that insomnia patients display characteristic gut microbiota changes, including elevated Bacteroidetes, reduced Firmicutes and Proteobacteria, and decreased Firmicutes-to-Bacteroidetes ratios compared to healthy individuals (Adak and Khan, 2019; Nagpal et al., 2018; Zhou et al., 2022). Animal and human shift worker studies confirm that sleep deprivation rapidly modifies gut microbiota diversity and composition. For example, Thaiss et al. (2016) noted that perturbing the sleep cycles of mice led to changes in the composition and diversity of their gut microbiota, and Reynolds et al. (2017) reported analogous findings in shift workers who were sleep-deprived. These alterations may influence sleep through several mechanisms: (1) microbial metabolites (tryptophan) supporting serotonin/melatonin synthesis; (2) SCFAs regulating blood–brain barrier function and clock genes; (3) LPS-induced neuroinflammation via TLR4/NF-κB signaling (Matenchuk et al., 2020; Wang et al., 2021).

Current evidence demonstrates that pro-inflammatory cytokines, particularly interleukin-1β (IL-1β) and tumor necrosis factor-α (TNF-α) in the central nervous system, play a crucial regulatory role in the sleep–wake cycle through specific neuroimmune signaling pathways (Imeri and Opp, 2009). Clinical observations reveal that patients with chronic insomnia exhibit significantly higher circulating levels of IL-1β compared to healthy controls, accompanied by a marked increase in pro-inflammatory bacterial taxa within their gut microbiota (Li et al., 2020). Mechanistic studies suggest that sleep deprivation initiates a cascade of inflammatory events that are substantially mediated by gut microbial communities (Matenchuk et al., 2020), with emerging data indicating that gut dysbiosis can simultaneously provoke both systemic (peripheral) and neuroinflammatory (central) responses—a breakthrough discovery that may lead to novel microbiota-targeted interventions for mitigating the detrimental consequences of sleep loss (Wang et al., 2021). In a separate study, Bowers et al. (2022) investigated the effects of a prebiotic mixture containing GOS and polydextrose (PDX) in mice. Their findings showed that sleep-deprived mice experienced extended non-rapid eye movement and rapid eye movement sleep durations. This suggests that GOS may improve sleep quality by modulating the gut microbiota. Considering the gastrointestinal tract’s dual function as both a primary site of immune activity and a key regulator of circadian processes, it is imperative that future investigations prioritize elucidating the complex interplay between gut-derived inflammation, host immune responses to microbial populations, and their collective influence on circadian rhythm modulation (Teichman et al., 2020).

However, current sleep-microbiota research encounters three primary methodological limitations that warrant careful consideration. First, the predominant reliance on subjective sleep assessments (e.g., PSQI questionnaires in Li et al., 2020; Li et al., 2020), demonstrates only partial concordance with objective polysomnography measurements (Smith et al., 2019). Second, critical confounding variables remain inadequately addressed across studies, as evidenced by Zhou et al.’s (2022) finding that 30% of participants used antidepressants without proper statistical adjustment (Zhou et al., 2022). Third, while 16S rRNA sequencing represents the dominant analytical approach (employed in 80% of existing literature), this technique fails to provide functional metabolic insights (Allaband et al., 2019). To address these constraints, integrating actigraphy-based sleep monitoring with metagenomic sequencing emerges as a promising methodological advancement for future investigations (Matenchuk et al., 2020).

The reciprocal interactions between sleep patterns and gut microbial communities highlight the pivotal role of modifiable lifestyle factors in preserving microbial equilibrium. While sleep patterns significantly modulate microbial communities, physical activity emerges as another key lifestyle factor that interacts bidirectionally with the gut microbiota. Regular physical activity serves as a potent modulator of this system, exerting beneficial effects through both enhancing microbial diversity and optimizing metabolic function.

### 3.3 Exercise and gut microbiota interactions

A growing body of evidence from human and animal studies demonstrates a complex bidirectional relationship between physical activity and gut microbial composition, mediated through multiple physiological pathways. While the precise molecular mechanisms remain under active investigation, current data suggest that exercise exerts dose-dependent effects on gut microbiota, with long-term interventions (>8 weeks) producing more robust and consistent increases in microbial α-diversity and enhanced production of beneficial metabolites including butyrate compared to acute exercise sessions (Mohr et al., 2020; Ortiz-Alvarez et al., 2020). Importantly, the interaction between exercise and dietary patterns appears to be synergistic, accounting for 40–60% of observed inter-individual microbial variations in athletic populations, with protein intake and fiber consumption being particularly influential modulators (Goodrich et al., 2014; Murtaza et al., 2019). These effects may be mediated through exercise-induced alterations in gut transit time, intestinal pH, and bile acid profiles, creating distinct ecological niches for microbial colonization (Goodrich et al., 2014; Knight et al., 2017).

Sport-specific microbial signatures have emerged as a particularly intriguing area of investigation. Endurance athletes (e.g., marathon runners, cyclists) consistently demonstrate 2–3 fold higher abundance of *Prevotella copri* and related species, which encode enhanced carbohydrate-active enzymes (CAZymes) for efficient energy harvest from complex polysaccharides (Jang et al., 2019; Mohr et al., 2020). In contrast, strength-trained athletes exhibit microbial communities enriched in proteolytic species (e.g., *Bacteroides* spp.) with upregulated peptidase activity (Jang et al., 2019). Notably, elite marathon runners show a remarkable 5–8 fold increase in *Veillonella atypica*, which converts exercise-induced lactate into propionate - a metabolic pathway shown to improve running endurance by 13–15% in murine models (Scheiman et al., 2019). Studies have demonstrated that high-intensity interval training (HIIT) more significantly improves peak VO₂ and alters microbial metabolites associated with insulin sensitivity compared to moderate-intensity continuous training (MICT) (Jiang et al., 2024; Kasperek et al., 2023). Cross-sectional comparisons reveal that professional athletes across disciplines (cyclists, rugby players, swimmers) exhibit 20–25% greater microbial diversity (Chao1 index) and enhanced functional capacity for amino acid and carbohydrate metabolism compared to sedentary controls (Barton et al., 2018; Clarke et al., 2014; Petersen et al., 2017). These differences persist after controlling for dietary variables, suggesting an independent effect of exercise training (Clarke et al., 2014).

At the mechanistic level, exercise-microbiota interactions operate through three well-characterized pathways: First, metabolic modulation occurs through increased abundance (2–4 fold) of mucin-producing *Akkermansia muciniphila*, which strengthens gut barrier integrity, and butyrate-generating *Roseburia hominis* (3–5 fold increase), which serves as a key regulator of colonic homeostasis (Hughes, 2019; Mohr et al., 2020). Second, immune system regulation is achieved through exercise-induced increases (30–40%) in anti-inflammatory cytokines (IL-10, TGF-β) and enhanced proliferation of regulatory T cells (Tregs), mediated by microbial antigens (Nieman and Wentz, 2019). Third, intestinal barrier function is enhanced through exercise-mediated alterations in bile acid metabolism, particularly increased secondary bile acid production (e.g., deoxycholic acid) which inhibits FXR signaling and reduces endotoxin translocation by 40–50% (Goodrich et al., 2014; Knight et al., 2017). These pathways collectively contribute to the observed improvements in metabolic health parameters (e.g., insulin sensitivity, lipid profiles) in regularly exercising individuals (Mohr et al., 2020; Ortiz-Alvarez et al., 2020).

Despite these significant advances, several critical methodological limitations must be addressed in future research. Current studies frequently conflate acute exercise effects (e.g., marathon-induced changes lasting 72 h) with chronic training adaptations (e.g., year-round rugby training) (Barton et al., 2018; Scheiman et al., 2019), and over 65% fail to adequately control for dietary variables - a major confounding factor given the tight diet-exercise interplay (Goodrich et al., 2014; Jang et al., 2019). Additionally, inconsistent findings regarding microbial diversity measures persist, with some studies reporting 20–30% increases in α-diversity (Clarke et al., 2014), while others show no significant changes (O’Donovan et al., 2020), possibly due to variations in sequencing depth (range: 20,000–100,000 reads/sample) and bioinformatic pipelines. To address these limitations, we recommend: (1) longitudinal study designs with pre/post-intervention assessments and standardized dietary controls; (2) integrated multi-omics approaches combining metagenomics, metabolomics and proteomics; and (3) sport-specific investigations with larger sample sizes (*n* > 100 per group) to account for inter-individual variability (Barton et al., 2018; Clarke et al., 2014; O’Donovan et al., 2020). Such methodological improvements will be essential for translating these findings into targeted microbiota-based interventions for both athletes and the general population.

Additionally, future research should focus on the effects of different types of exercise (e.g., aerobic exercise, strength training, flexibility training) on gut microbiota. Studies should consider the long-term (>8 weeks) and short-term (<8 weeks) effects of various exercise intensities (e.g., low, moderate, and high intensity) to determine the specific impacts of each exercise type and intensity on the microbiota. For example, endurance exercise (such as marathon running, prolonged cycling) may promote the proliferation of microbes like *Prevotella*, while strength training (such as weight lifting, short high-intensity interval training) may increase the abundance of proteolytic bacteria like *Bacteroides* spp. Furthermore, research should explore the long-term effects of different exercise durations (e.g., weekly exercise hours) on microbial diversity and metabolic function, especially in different age groups (such as older adults) and specific health conditions (e.g., obesity, diabetes). These studies will help develop personalized exercise and dietary intervention plans for different populations, maximizing the benefits of exercise on gut health.

## 4 Current research gaps and future directions

The field of lifestyle-microbiota interactions faces three fundamental challenges that hinder translational applications. First, methodological heterogeneity persists across studies, with substantial variations in exercise protocols (type/frequency/intensity), dietary monitoring approaches (FFQs vs. controlled feeding), and sequencing techniques (16S rRNA vs. metagenomics) - exemplified by 5-fold differences in reported Prevotella enrichment among endurance athletes due to sequencing depth disparities (20,000–100,000 reads/sample). Second, uncontrolled confounders (e.g., circadian disruptions, medication use) introduce significant noise, as evidenced by Zhou et al.’s finding that 30% of sleep studies failed to account for antidepressant use. Third, technological limitations prevail, where 80% of existing literature relies on 16S rRNA sequencing that lacks functional resolution, while inconsistent bioinformatic pipelines yield conflicting α-diversity results (20–30% increases vs. null effects). These issues are compounded by frequent conflation of acute exercise responses (e.g., 72 h post-marathon changes) with chronic adaptations (year-round training effects), and inadequate sample sizes (*n* < 30 in 45% of diet-microbiota studies) that limit statistical power.

To overcome these barriers, we propose a tripartite roadmap for next-generation research: (1) Standardized protocols incorporating ISO-certified exercise regimens, validated dietary tracking tools (ASA24), and unified multi-omics workflows (prioritizing metagenomics for functional insights); (2) Large-scale longitudinal randomized controlled trials (RCTs) (n > 100/group) with stringent control of host variables (age, BMI, medication) and integrated actigraphy-microbiota monitoring to disentangle circadian effects; (3) Mechanistic synergy studies employing germ-free models and fecal microbiota transplantation to investigate how diet-exercise-sleep combinations (e.g., high-fiber diets + endurance training) modulate specific microbial functions (e.g., *Roseburia-*mediated butyrate production). Such approaches should be complemented by cross-validation of sequencing platforms (Illumina vs. Nanopore) and establishment of microbial “responder” thresholds (e.g., >10% *Faecalibacterium* increase) to enhance reproducibility.

Critical knowledge gaps demanding urgent attention include: (1) Exercise-type specificity- resolving how resistance training preferentially enriches proteolytic *Bacteroides* versus aerobic exercise-induced *Prevotella* through microbial lactate metabolism; (2) Population-tailored dynamics - determining optimal lifestyle prescriptions for elderly (probiotics + protein supplementation) versus metabolic syndrome patients (high-fiber diets + moderate exercise) to counteract age- or disease-related dysbiosis; (3) Circadian-microbiota crosstalk - elucidating how microbial metabolites (SCFAs, tryptophan) regulate clock genes in shift workers. As highlighted in Table 3, addressing these priorities through concerted multidisciplinary efforts will enable development of precision microbiota interventions targeting immune-metabolic disorders, bridging the current gap between mechanistic insights and clinical applications. Future studies should particularly focus on longitudinal monitoring of athlete cohorts and high-risk populations (T2DM, elderly) to establish causal timelines for microbial changes and their functional health impacts.

**Table 3:** Critical appraisal of microbiota research limitations and future directions across lifestyle domains.

Domain	Major limitations	Conflicting findings	Proposed solutions	Priority research gaps
Diet	Small sample sizes Cross-sectional designs Inconsistent findings	Firmicutes: Bacteroidetes ratio changes inconsistent (↑ in 60% vs. ↔ in 40% of high-protein diet studies) Mediterranean diet effects on α-diversity (↑ in 65% vs. ↔ in 35% of trials)	Standardized 7-day weighed food records + biomarkers Longitudinal RCTs with multi-omics Baseline microbiota stratification	MACs-microbiome dose–response Host genetics-microbiota interactions Personalized nutrition algorithms
Sleep	Overreliance on subjective sleep measures (PSQI) 30% studies fail to control antidepressant use 80% studies use 16S rRNA without functional data	55% vs. 45% studies report microbial diversity changes in insomnia Bidirectional causality debates (microbiota→sleep vs. sleep→microbiota)	Actigraphy + polysomnography integration Medication-naïve cohort studies Metagenomics + metabolomics pairing	Microbial circadian rhythms LPS-neuroinflammation axis FMT timing effects
Exercise	Diet-exercise confounding (65% of studies) Intensity quantification lacking (70% of trials) Homogeneous populations (85% young athletes)	Inconsistent reports on microbial diversity: some show 20–30% ↑ α-diversity, others find no change, possibly due to sequencing depth (20,000–100,000 reads/sample) or bioinformatics differences	Longitudinal designs + standardized diet control Multi-omics integration (metagenomics, metabolomics, proteomics) Sport-specific studies with larger cohorts (*n* > 100/group)	Compare exercise types (aerobic/strength/flexibility) on microbiota Dose–response: intensity (low/moderate/high) and duration (short- < 8w, long- > 8w) Population-specific effects (age, obesity/diabetes) Mechanisms: e.g., endurance→↑*Prevotella*; strength→↑*Bacteroides*

↑ indicates increase; RCTs, randomized controlled trials; MACs, microbiota-accessible carbohydrates; PSQI, Pittsburgh sleep quality index; LPS, lipopolysaccharide; FMT, fecal microbiota transplantation.

## 5 Summary and translational perspectives

Collectively, the interplay of dietary habits, sleep architecture, and exercise regimens shapes gut microbial ecosystems in distinct yet interconnected ways, ultimately influencing host health outcomes. Having addressed the key research gaps and methodological challenges, this comprehensive synthesis establishes the gut microbiota as a pivotal mediator linking modifiable lifestyle factors (diet, sleep, and exercise) to host physiology and disease susceptibility. Our analysis demonstrates that targeted lifestyle interventions can reshape microbial communities to favor beneficial taxa (e.g., fiber-fermenting *Roseburia* and *Faecalibacterium*) while suppressing pro-inflammatory species, with measurable impacts on metabolic, immune, and neurological health outcomes. The accumulated evidence positions microbiota modulation as a promising strategy for chronic disease prevention and management, particularly for conditions like metabolic syndrome, IBD, and neurodegenerative disorders where dysbiosis plays an established pathogenic role.

Moving forward, the field must transition from observational correlations to mechanistic, causal understandings through: (1) standardized multi-omics protocols that resolve functional pathways beyond taxonomic profiling; (2) large-scale longitudinal interventions controlling for key confounders (genetics, medications, circadian rhythms); and (3) personalized approaches accounting for interindividual microbial variability. The integration of these strategies with emerging technologies - including AI-driven microbiota analysis and wearable monitoring devices - will accelerate the development of precision microbiota medicine. These advances promise to transform public health paradigms by enabling evidence-based, microbiota-conscious lifestyle recommendations tailored to individual risk profiles and health statuses.
